# Female Reproductive Tract Modeling through Advanced 3D Biomimetic Platforms

**DOI:** 10.34133/bmr.0413

**Published:** 2026-09-03

**Authors:** Chaeyoun Lee, Yoonseo Choi, Hyejin Lee, Seunghee Kim, Jeong Su Park, Young Min Shin, Dae Hyeok Yang, Gun-Jae Jeong, Dong Nyoung Heo, Jeong-Kee Yoon, Junhyeong Yim, Jongmin Kim, Yonghwan Kim, Kyung Hyun Yoo, Jung Bok Lee, Byung-Chul Lee

**Affiliations:** ^1^ Department of Biological Sciences, Sookmyung Women’s University, Seoul 04312, Republic of Korea.; ^2^ Research Institute of Women’s Health, Sookmyung Women’s University, Seoul 04312, Republic of Korea.; ^3^ PLCOskin Co. Ltd., Seoul 03721, Republic of Korea.; ^4^ Department of Biologics and Engineering, The Catholic University of Korea, Bucheon-si 14662, Gyeonggi-do, Republic of Korea.; ^5^ School of Nanomedical Engineering, Korea National University of Transportation, Chungju 27469, Republic of Korea.; ^6^ Department of Dental Materials, School of Dentistry, Kyung Hee University, Seoul 02447, Republic of Korea.; ^7^ Department of Systems Biotechnology, Chung-Ang University, Anseong-Si, Gyeonggi-Do 17546, Republic of Korea.

## Abstract

The female reproductive tract (FRT) is an intricate and highly regulated network comprising the ovaries, fallopian tubes, uterus, cervix, and placenta, which work in concert to govern complex reproductive functions. Dysfunction within any of these organs can lead to serious pathological conditions, including infertility, pregnancy complications, and gynecological cancers. Although conventional 2-dimensional (2D) cultures and animal models have provided foundational insights, they are limited in their ability to recapitulate human-specific 3D tissue architecture and dynamic biochemical microenvironments. To address these limitations, organoid and organ-on-a-chip (OoC) technologies have emerged as a powerful 3D biomimetic platform. Organoids preserve epithelial identity, cellular heterogeneity, and patient-specific phenotypes, whereas OoC systems incorporate microfluidic flow, mechanical stimulation, and multicompartmental interfaces to model organ-level physiology. This review provides an organ-specific overview of recent advances in organoid and OoC systems across the FRT, discusses their advantages and current limitations relative to traditional models, and highlights their potential to transform reproductive biology research, disease modeling, and translational applications.

## Introduction

The female reproductive tract (FRT) is a complex and highly orchestrated biological system essential for maintaining reproductive capacity. It governs a precise sequence of events, including ovulation, fertilization, embryo and fetal development, and ultimately parturition. The successful execution of these processes relies on the tight structural and physiological coordination of individual organs, including the ovaries, fallopian tubes, uterus, cervix, and placenta [1]. Traditionally, our understanding of these mechanisms and their associated pathologies has largely relied on murine models and conventional 2-dimensional (2D) cell cultures. While these traditional platforms have provided foundational insights, particularly regarding folliculogenesis, endocrine signaling, and reproductive disorders [2], their translational relevance to human biology is often constrained by inherent species-specific discrepancies in genetics, anatomy, and hormonal regulation [3,4]. Nonhuman primate (NHP) models partially bridge this gap, offering closer anatomical resemblance to the human FRT, but remain limited by ethical, financial, and technical constraints. Similarly, while standard 2D cultures offer experimental simplicity and cost-effectiveness, they fail to reproduce the intricate 3D architecture and dynamic biochemical milieu characteristic of the native in vivo environment [5].

**Fig. 1.**
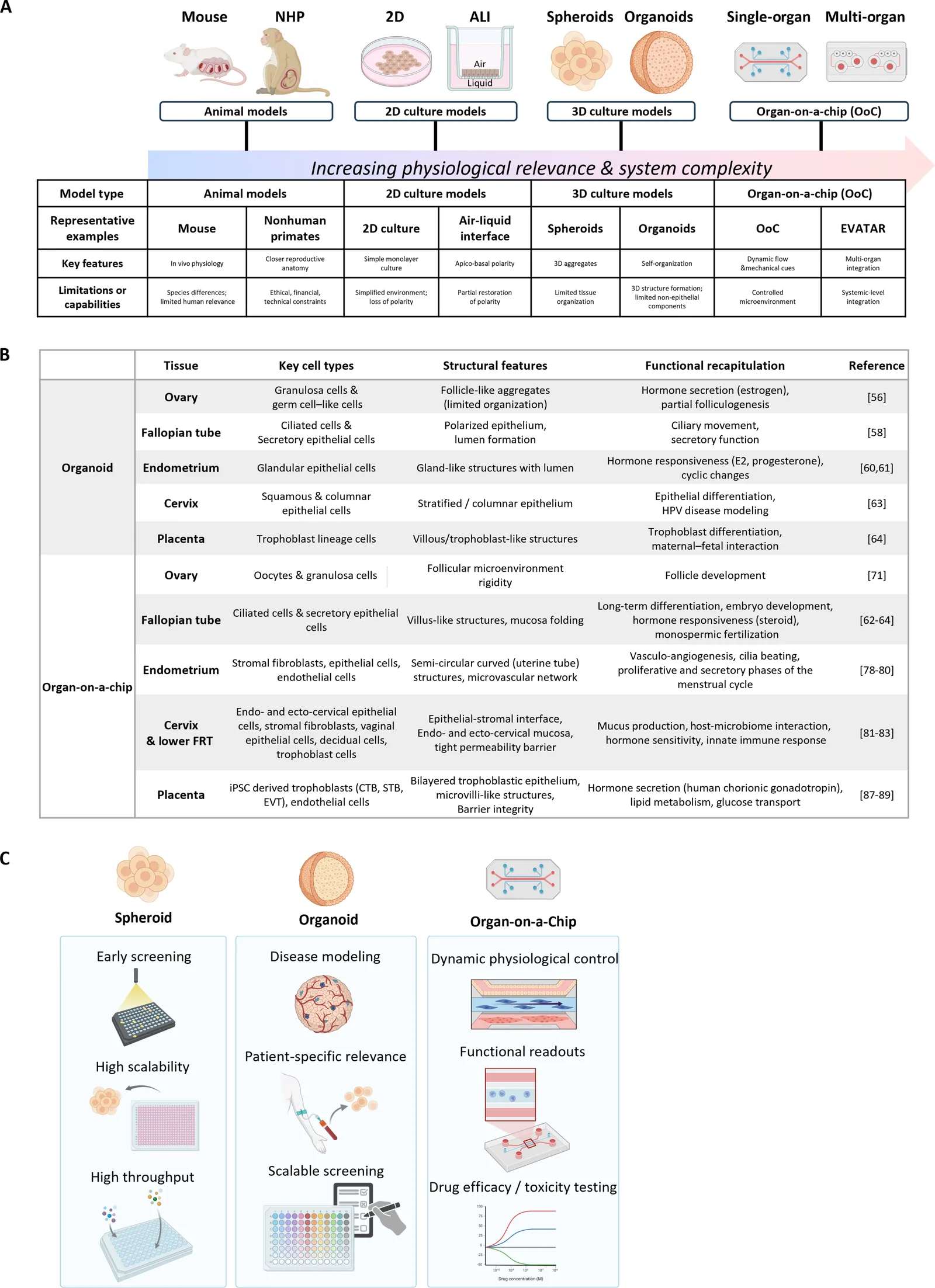
Evolution and functional features of female reproductive tract (FRT) models. (A) Experimental systems have progressed from in vivo animal models to advanced in vitro platforms, including 2-dimensional (2D) cultures, air–liquid interface (ALI) systems, spheroids, organoids, and organ-on-a-chip (OoC) technologies. Each step improves the ability to recapitulate human-specific physiology, structural complexity, and dynamic microenvironmental cues. (B) Organoid and OoC models representing the FRT tissues recapitulate structural features and physiological functions. CTB, cytotrophoblast; STB, syncytiotrophoblast; EVT, extravillous trophoblast. (C) Advantages and characteristics of spheroids, organoids, and OoC systems.

To bridge the gap between conventional in vitro models and human physiology, 3D models, including organoids and organ-on-a-chip (OoC) systems, have been developed. Organoids, which utilize human-derived cells, offer substantial advantages over animal models, providing species-specific physiology and capturing human-relevant cellular, genetic, and molecular features. They also enable long-term culture, patient-specific modeling, and high-throughput experimentation, making them a powerful platform for studying human reproductive biology [6]. Complementing these 3D constructs, OoC platforms have emerged as biomimetic systems capable of replicating organ-level functions within a precisely controlled microenvironment. Unlike static cultures, these microfluidic devices apply mechanical and biochemical cues to emulate physiological processes [7]. By integrating multiple cell types across permeable membranes, these systems reproduce the structural and functional heterogeneity of native barriers, features that remain unobtainable in conventional models [8]. Consequently, reproductive OoC models represent a major step toward physiologically relevant human systems, enabling dynamic investigation of organ-level reproductive processes under controlled conditions (Fig. 1A and B).

In this review, we summarize the evolution of experimental models for the female reproductive system, ranging from conventional animal and 2D culture models to organoids and OoC systems. Unlike previous reviews that have generally focused on individual reproductive organs or examined specific model platforms separately, this review integrates recent advances across the ovary, fallopian tube, uterus, cervix, and placenta within a unified framework of 3D new approach methodologies (NAMs). By directly considering both organoid and OoC technologies, we highlight their complementary strengths, current technical and biological limitations, and translational potential. This comprehensive perspective provides a broader understanding of how these models can advance reproductive biology, disease modeling, drug screening, toxicity assessment, and the development of more physiologically relevant preclinical platforms.

## Anatomy and Physiology of the Female Reproductive Organs

### Ovary

The ovary is central to female reproduction and endocrine homeostasis, serving both as a reservoir of follicles and as the principal source of sex hormones, including estrogen and progesterone. Structurally, each ovary consists of an outer cortex, which harbors follicles at various stages of development, and an inner medulla containing vascular and connective tissue. Functionally, ovarian reserve is defined by the size and activity of the intra-ovarian population of growing follicles, which progress through primordial, primary, secondary, antral, and preovulatory stages, from which a single dominant follicle is typically selected for ovulation in each cycle. Anatomically, the paired ovaries lie within the pelvic cavity near the lateral pelvic wall and are attached to the uterus by the mesovarium, a double fold of peritoneum that provides structural support and serves as a conduit for the vascular and lymphatic supply [9].

Throughout the female lifespan, ovarian morphology and function undergo dynamic remodeling driven by reproductive stage and individual physiology. Ovarian volume, determined by the integrated mass of stromal tissue and follicles, varies significantly. It is relatively small and endocrinologically quiescent during prepubertal and post-reproductive periods, whereas it exhibits hypertrophy and heightened steroidogenic activity from puberty to menopause [10]. Notably, ovarian size can increase by more than double during pregnancy, highlighting the organ’s plasticity in response to systemic hormonal cues [9]. Surface morphology also reflects reproductive history: While smooth in early life, repeated cycles of ovulation and subsequent corpus luteum formation result in cumulative scarring, producing a progressively irregular surface [11]. These features collectively illustrate the continuous tissue remodeling that links folliculogenesis to endocrine function throughout a woman’s reproductive lifespan. This dynamic relationship between structural plasticity and hormonal output stresses the complexity of the ovarian microenvironment and the challenges inherent in replicating it in vitro.

### Fallopian tube

The fallopian tube is a key conduit within the FRT, enabling gamete transport and constituting the principal site of fertilization and early embryo development. Measuring approximately 7.6 to 10.5 cm in length, it links the ovary to the uterine cavity, with length and caliber varying across adolescence, the reproductive years, and the postmenopausal period [12]. The tube is divided into 4 contiguous segments: the intramural (interstitial) section, isthmus, ampulla, and the infundibulum equipped with fimbriae [13]. These regions exhibit distinct topographical and epithelial profiles, particularly regarding the distribution of multi-ciliated cells, which are highest distally and decrease toward the uterine interface [14]. The intramural segment is the narrowest portion traversing the myometrium (uterotubal junction) and, normally a passageway into the uterus, is a recognized site for rare interstitial ectopic implantation. The isthmus contains fewer ciliated cells than the ampulla and acts as a functional reservoir and regulator of gamete/embryo transit. Fertilization primarily occurs within the ampulla. The infundibulum and its fimbriae abut the ovary; their richly ciliated epithelium and mobile fimbrial fringe are specialized to capture the ovulated oocyte [15,16].

Structurally, the tubal wall comprises 3 concentric layers: an outer serosa (mesosalpinx), a middle smooth-muscle layer (myosalpinx), and an inner mucosa (endosalpinx) with prominent longitudinal folds [17]. The serosa reflects the visceral peritoneum. The myosalpinx contains interlacing circular and longitudinal fibers that generate peristaltic activity. The endosalpinx is lined by a pseudostratified epithelium composed of ciliated, secretory, peg (intercalated), and basal cells. Coordinated ciliary beating within the endosalpinx, together with myosalpinx peristalsis, propels gametes and preimplantation embryos toward the uterine cavity. Both the tubal epithelium and musculature are highly responsive to the cyclic gonadotropin-steroid dynamics of the menstrual cycle [18,19]. During the follicular phase, rising estradiol augments ciliogenesis and ciliary beat frequency, enhances secretory activity, and increases fimbrial motility and apposition to the ovary, thereby optimizing oocyte pickup and distal-to-proximal transport [20,21]. Following ovulation, the mid-cycle luteinizing hormone (LH) surge triggers corpus luteum formation, after which progesterone partially down-modulates ciliary activity and alters secretory profiles while modifying smooth-muscle contractility, creating a microenvironment favorable to embryo transit toward the uterus. These cyclic, segment-specific adjustments in epithelial phenotype and motor function underpin the spatiotemporal coordination of fertilization and early embryonic passage.

### Uterus

The uterus is a thick, fibromuscular organ situated within the female pelvis between the urinary bladder and rectum, providing the essential environment for implantation, gestation, and parturition. Morphologically, it is pear-shaped and anteroposteriorly flattened, with a relatively planar anterior surface and a more convex posterior surface [22]. It is conventionally divided into the uterine body (corpus) superiorly and the cervix inferiorly. The uterine wall comprises 3 functionally distinct layers: the endometrium, a mucosa lining the cavity that undergoes cyclic proliferation, differentiation, and shedding to permit implantation; the myometrium, the thick smooth-muscle layer interspersed with collagen that generates the contractile forces of labor; and the perimetrium, the serosal covering of the intra-abdominal portion [23].

The endometrium is functionally organized into 2 distinct layers: the superficial functional layer, which undergoes cyclic estrogen- and progesterone-driven proliferation, secretory transformation, and menstrual shedding, and the deeper basal layer, which serves as a regenerative reservoir following menstruation [24]. During the proliferative phase, estrogen stimulates glandular and stromal growth; during the secretory phase, progesterone drives glandular secretion and stromal decidualization to establish the implantation window [25]. In the absence of a fertilized embryo, progesterone withdrawal triggers menstruation and renewal of the cycle. The myometrium similarly exhibits hormonal responsiveness, with progesterone maintaining uterine quiescence during pregnancy and oxytocin-mediated contractions driving parturition at term.

Uterine dimensions vary with age, parity, and hormonal milieu. In nulliparous adults, the uterus typically measures 7 to 8 cm in length, whereas parous uteri are generally larger owing to pregnancy-associated hypertrophy and only partial involution postpartum. After menopause, hypoestrogenism is associated with progressive reduction in uterine size and an atrophic endometrial appearance [26].

### Cervix

The uterine cervix is a dynamic, fibroconnective organ that serves as the gateway between the uterine cavity and the vagina, fulfilling distinct mechanical, secretory, and barrier functions across the reproductive lifespan. During pregnancy, it maintains a closed, mechanically competent canal to support fetal retention; at term, it undergoes profound remodeling to become a compliant birth passage permitting safe neonatal delivery.

Cervical tissue is predominantly fibrous connective tissue organized around 2 broad components: an extracellular matrix (ECM) rich in collagen, with contributory elastin and proteoglycans, and a cellular compartment comprising smooth-muscle cells, fibroblasts, epithelial cells, and the vascular bed. These constituents are distributed heterogeneously along the cervix. The distal (vaginal) segment contains a higher proportion of ECM and relatively less smooth muscle, whereas the proximal region adjoining the uterine myometrium contains comparatively more muscle [27,28]. This longitudinal compositional gradient provides the mechanistic basis for the cervix’s biphasic mechanical program: sustained stiffness and occlusion throughout gestation, followed by precisely regulated compliance and dilation at term.

Over the course of gestation, the cervix transitions through a phased program of viscoelastic remodeling, encompassing softening, ripening, effacement, and dilation, that enables fetal expulsion and triggers postpartum reparative processes. Early in gestation, the cervix is elongated and firm, but as term approaches, it progressively softens and shortens. Insufficient cervical remodeling despite adequate uterine contractions can lead to labor arrest, whereas premature remodeling predisposes to cervical insufficiency and preterm birth [29]. Collectively, these temporally regulated, region-specific changes in ECM architecture and cellular activity are central determinants of labor onset and birth timing.

### Placenta

The placenta is a transient, highly specialized organ that develops from extraembryonic tissues early in gestation and is indispensable for fetal growth and survival. Its development and function govern the intrauterine milieu, influencing gas exchange, nutrient delivery, waste removal, immune tolerance, and endocrine signaling throughout pregnancy. Placental development is initiated by the implantation of the blastocyst into the uterine endometrium, followed by trophoblast differentiation into 3 functionally distinct subtypes: cytotrophoblasts (CTBs), which serve as proliferative progenitor cells; syncytiotrophoblasts (STBs), which form the multinucleated epithelial layer directly interfacing with maternal blood; and extravillous trophoblasts (EVTs), which invade the decidua and remodel uterine spiral arteries to establish adequate uteroplacental blood flow [30].

In humans, placentation is hemochorial and villous: Maternal blood in the intervillous space directly contacts the apical surface of the STB investing the chorionic villi [30]. Architecturally, a branching villous tree arises from the chorionic plate into the intervillous space: Large stem villi provide structural support, intermediate villi ramify from these trunks, and terminal villi constitute the principal sites of materno–fetal exchange [31]. This hierarchical arborization substantially expands exchange surface area, enabling diffusion of respiratory gases and carrier-mediated transport of nutrients and solutes, while fetal capillaries within the villous core minimize diffusion distance.

Beyond exchange functions, the placenta is a major endocrine organ that secretes hormones into the maternal and fetal circulations, thereby modulating maternal metabolism and supporting fetal development. Key products include chorionic gonadotropin, placental lactogen/placental growth hormone, progesterone, and estrogens, which sustain luteal function early, modulate insulin sensitivity and substrate availability, and coordinate maternal organ adaptations until parturition [32].

## Traditional Models for Studying Female Reproductive System

### Animal models

Animal models have long served as the cornerstone of reproductive biology research, providing physiologically intact systems in which to investigate hormonal regulation, organ-level interactions, and disease mechanisms. Among these, murine models are the most widely employed, owing to their well-characterized genetics, short reproductive cycles, high fecundity, and amenability to genetic manipulation. In vivo murine models have been used to interrogate a broad range of reproductive processes, including folliculogenesis, oviductal cilia formation and function, endometrial remodeling, and implantation. Both pathological and physiologically normal states have been modeled in mice [33]. Notably, Greaves et al. [2] established a menstrual endometrium model by intraperitoneally transplanting menstrual endometrium into immunocompetent mice, producing lesions and estrogen-responsive phenotypes reflecting the human condition. Nevertheless, important interspecies differences limit the translational fidelity of murine models. Of note, humans are mono-ovulatory with oocytes concentrated within cortical pockets, whereas mice are poly-ovulatory with a more homogeneous oocyte distribution [3]. Although both species have a single cervix, mice have a bicornuate uterus and highly coiled oviducts, and lack spontaneous decidualization or true menstruation [34,35]. These anatomical and physiological discrepancies substantially limit the direct extrapolation of murine findings to human reproductive biology.

To bridge the gap between murine models and human biology, NHP models have been employed, offering closer anatomical, hormonal, and physiological resemblance to humans. Rhesus macaques and baboons, for instance, share spontaneous menstruation, similar ovarian cycle dynamics, and hemochorial placentation with humans, making them valuable for studying endometriosis, implantation, and early pregnancy. Adult female marmosets possess a single fused uterus with uncoiled, gently arched fallopian tubes that more closely approximate human reproductive tract architecture [36]. However, reproductive research by using NHPs remains constrained by substantial ethical, financial, and technical barriers. Therefore, there remains a need for in vitro platforms that more faithfully reconstruct human reproductive tract complexity with great fidelity, thereby enabling rigorous target validation and therapeutic testing without reliance on cross-species extrapolation.

### 2D cell culture models

Despite their inability to replicate native tissue complexity, the conventional 2D culture systems have remained the predominant models due to their simplicity and cost-effectiveness. Across the FRT, 2D cultures have been established from a variety of epithelial cell types, including those derived from the oviduct, endometrium, cervix, and placenta, each providing accessible platforms for investigating cell-specific physiology. Although advances in optimizing ex vivo primary cell culture conditions have expanded the utility of 2D culture systems, primary cells still exhibit inherent limitations, including restricted proliferative capacity and significant interindividual variability. To address these limitations, Deng et al. [37] isolated epithelial cells from the ectocervix, transformation zone, and endocervix, and systematically compared proliferation and colony-forming capacity to identify optimal culture condition. This work culminated in a stepwise protocol for reliable cervical epithelial cell culture.

To more accurately recapitulate in vivo epithelial environments in FRT, compartmentalized systems such as the air–liquid interface (ALI) have been established as an intermediate approach between conventional 2D monolayers and fully 3D constructs [38,39]. Under ALI conditions, endometrial epithelial cells acquired in vivo-like structural features, including cilia, microvilli, and secretory granules, while exhibiting hormone responsiveness and the ability to differentiate into stromal-like cells. ALI culture of the oviduct epithelium facilitated the characterization of epithelial interactions with gametes and early embryos in molecular level [40]. 2D cultures of bovine oviduct epithelial cells (BOECs) have also been widely used to investigate oviductal functions in sperm selection, viability, and release [41]. Jordaens et al. [5] comprehensively compared BOEC monolayers, explants, and hanging inserts to determine conditions that best preserve epithelial polarity. The results showed that the hanging insert system divided the cells into 2 compartments and enabled unidirectional or bidirectional exposure, mimicking in vivo environment. Similarly, Huo et al. [42] demonstrated that culturing porcine oviduct epithelial cells (POECs) under ALI conditions enhanced epithelial differentiation in an oxygen-dependent manner. Extending this approach to feline models, Eder et al. [43] reported that long-term differentiated cultures of feline oviduct epithelial cells (FOECs) maintained stable epithelial phenotypes for over 3 weeks. Although their stability is limited and cannot be sustained over extended culture periods, these studies highlight that monolayer-based systems can reproduce key features of the oviduct epithelium, including polarized secretory and ciliated cells.

Beyond single epithelial lineage-ALI system, multicellular coculture systems have been developed to reproduce the cellular complexity of in vivo environments. For instance, coculturing mouse embryoid bodies derived from embryonic stem cells (ESCs) with granulosa cells promoted the differentiation of ESCs into oocyte-like cells (OLCs) [44]. Subsequent studies substituted ESCs with ovarian stem cells (OSCs), which similarly generated OLCs when cocultured with granulosa cells [45]. Although both approaches successfully induced oocyte-specific gene expression, they were unable to generate mature oocytes or organized follicular structures, remaining arrested at early developmental stages [44,45]. To recapitulate the placental barrier, trophoblast (BeWo b30) and fetal endothelial (HPEC-A2) cells have been cocultured and subsequently determined the permeability using tracers of varying sizes such as sodium fluorescein, fluorescein isothiocyanate–dextran, antipyrine, indomethacin, and nanoparticles [46].

While 2D culture models remain convenient and widely accessible, their inherent limitations render them insufficient to recapitulate the structural organization and physiological functions observed in vivo. Conventional 2D epithelial cultures often exhibit aberrant morphology and loss of apico-basal polarity, limiting their capacity to reproduce the ciliary dynamics, luminal fluid flow, stromal signaling, and immune-epithelial crosstalk. In particular, the absence of dynamic flow reduces the physiological relevance, suggesting the necessity for microfluidic refinement.

## Multicellular Constructs

### Spheroids

Among 3D platforms, ovarian follicle mimicking systems serve as promising alternatives to model native follicular niche [47]. Notably, a 3D oocyte spheroid comprising theca cells, granulosa cells, and oocytes successfully supported the maturation of early antral follicle oocytes to the metaphase II stage, representing the first successful application of tissue-engineering principles to oocyte maturation in vitro [47]. Beyond ovarian systems, several 3D spheroid models have been established to reconstruct the physiological and pathological microenvironments of the FRT. Lawrenson et al. [48] developed spheroid cultures using primary fallopian tube secretory epithelial cells (FTSECs), which more closely mirrored the molecular and cellular characteristics of native tissue than conventional monolayers. Within these spheroids, cells formed central hyaline cores surrounded by mono- or multilayered epithelial sheets, providing a physiologically relevant model for studying fallopian tube-related disorders and early events in ovarian carcinogenesis. Similarly, endometriotic spheroids (ES) containing epithelial, stromal, and peritoneal mesothelial cells have been created to reproduce key pathological features of endometriosis. Using this model, the study showed that proinflammatory macrophages increase spheroid invasion and that estradiol enhances invasion while progestin suppresses it [49]. Cervical-cancer spheroids derived from HeLa, CaSki, and SiHa cell lines have also been produced using the liquid-overlay method, exhibiting invasive behavior within the ECM [50].

### Ovarian organoid

Beyond spheroids, organoid systems enable long-term self-organization of complex structures and offer powerful tools for modeling tissue physiology and precision medicine (Fig. 1C and Fig. S1). The first human ovarian organoid was recently established by combining granulosa-like cells (GLCs) with human primordial germ-cell-like cells (hPGCLCs) with coexpression of *NR5A1* with either *RUNX1* or *RUNX2* identified as the minimal factors to induce GLC differentiation from induced pluripotent stem cells (iPSCs) [51]. These GLCs supported hPGCLC development, and the ovaroids demonstrated steroidogenesis, producing both estradiol (in the presence of androstenedione) and progesterone under distinct culture conditions, and the organoid recapitulated crucial ovarian phenotypes such as hormonal signaling, germ-cell maturation, and follicle formation, facilitating studies of human ovarian biology. Beyond organoid platforms, complementary approaches for modeling gynecological malignancies are also being pursued. For example, CD109, a cell surface glycoprotein, has been identified as a marker secreted in soluble and exosomal forms specifically from cancer stem-like cell (CSC) populations in ovarian cancer, suggesting its utility as a diagnostic and prognostic biomarker [52]. Integration of such CSC-derived biomarkers with patient-derived organoid systems would further enhance their value for precision oncology applications.

### Fallopian tube organoids

Fallopian tube organoids (FTOs) have reportedly demonstrated long-term stability for over 10 months, supporting epithelial stem cell populations within the fallopian tube (FT) epithelium. FT or oviductal organoids containing both secretory and ciliated cells also exhibit hormonal responsiveness in a physiological manner [53]. Lawson et al. [54] further established oviductal organoids from epithelial cells of 5 domestic species—bovine, porcine, equine, feline, and canine. These cross-species models closely resembled the native oviductal epithelium and have advanced our understanding of oviductal physiology while improving the potential of assisted reproductive technologies (ARTs).

### Endometrial organoids

Turco et al. [55] pioneered a long-term cultivable endometrial organoid (EMO) model derived from human endometrial tissue. The organoids exhibited continuous in vitro expansion in a chemically defined medium containing epidermal growth factor (EGF), Noggin, R-spondin-1, fibroblast growth factor 10 (FGF10), hepatocyte growth factor (HGF), A83-01, and nicotinamide. Under maturation-inducing conditions with estrogen, progesterone, and adenosine 3′,5′-monophosphate (cAMP), they differentiated into both secretory and ciliated epithelial cells. Furthermore, exposure to pregnancy-associated hormones, including human chorionic gonadotropin (hCG), human placental lactogen (hPL), and prolactin (PRL), induced features of early gestational endometrium. In parallel, Boretto et al. [56] established a robust and stable EMO system that recapitulated glandular organization, expressed lineage-specific markers, and displayed hormonally regulated proliferative, secretory, and withdrawal phases analogous to those of the menstrual cycle. Extending this cross-species approach, EMOs have been successfully established from endometrial epithelial stem cells of 5 mammalian species representing distinct placental morphologies including cows, dogs, cats, pigs, and rats, and maintained stably for over 13 passages. Transcriptomic analysis of these organoids highlighted conserved pathways such as phosphatidylinositol 3-kinase (PI3K)–Akt signaling and ECM–receptor interactions, along with significant hormone metabolism gene activity, stressing their potential as broadly applicable platforms for uterine physiology research and disease modeling across species [57].

### Cervix and placenta organoids

Long-term expandable endocervical and ectocervical organoids have been established from both normal and malignant cervical tissues [58]. Herpes simplex virus type 1 (HSV-1) infection assays revealed that these organoids reproduce key morphological and functional characteristics of their tissues of origin, providing a physiologically relevant platform for disease modeling, human papillomavirus (HPV) oncogenesis research, and patient-derived tumoroid-based drug screening. In parallel, trophoblast stem cell-derived organoids were generated to model key aspects of placental development. This system comprises spherical organoids featuring a single outer layer of STB cells with barrier function and a column-type STB barrier model reflecting epithelial organization [59]. These trophoblast organoids provide a useful experimental platform for studying early placental development and evaluating transplacental compound transfer and drug toxicity.

## Challenges and Future Advances in Organoids

Although organoids recapitulate key features of human tissue architecture and function, they remain subject to several important limitations. A major limitation is their incomplete cellular composition, as most tissue-derived organoids are predominantly epithelial and lack stromal, immune, neuronal, and vascular elements. This cellular simplicity limits their capacity to model mucosal interactions and physiological complexity observed in vivo [35]. In line with this, a limitation of ovarian organoids is the absence of androgen-producing theca cells. Although *NR2F2^+^* stromal-like cells can partially differentiate, these cells lack genuine theca cell function, and DAZL^+^ germ cells could not be maintained long-term [51]. While organoid systems offer distinct advantages over conventional 2D and animal models, they still fall short of reproducing the precise multicellular organization, intercellular communication, and dynamic microenvironment that define native reproductive organs [6]. To address their limited cellular diversity, organoids should be developed into more complex coculture systems or assembloids that incorporate additional cell types and more accurately recapitulate tissue-level interactions. Another major limitation is batch-to-batch variability arising from differences in culture protocols and operator-dependent procedures, which can compromise reproducibility. Moreover, conventional organoids lack dynamic physiological cues, including fluid flow and associated shear stress. Integrating organoids with OoC platforms may overcome this limitation by providing controlled perfusion and compartmentalized microenvironments. For example, patient-derived EMOs were dissociated into single cells and embedded within a hydrogel in an OoC system. Within this platform, the cells formed a polarized endometrial epithelial barrier, enabling the evaluation of barrier function, which is difficult to assess in conventional 3D organoids because of their closed architecture and potential formation of necrotic cores [60].

### Organ-on-a-chip

**Fig. 2.**
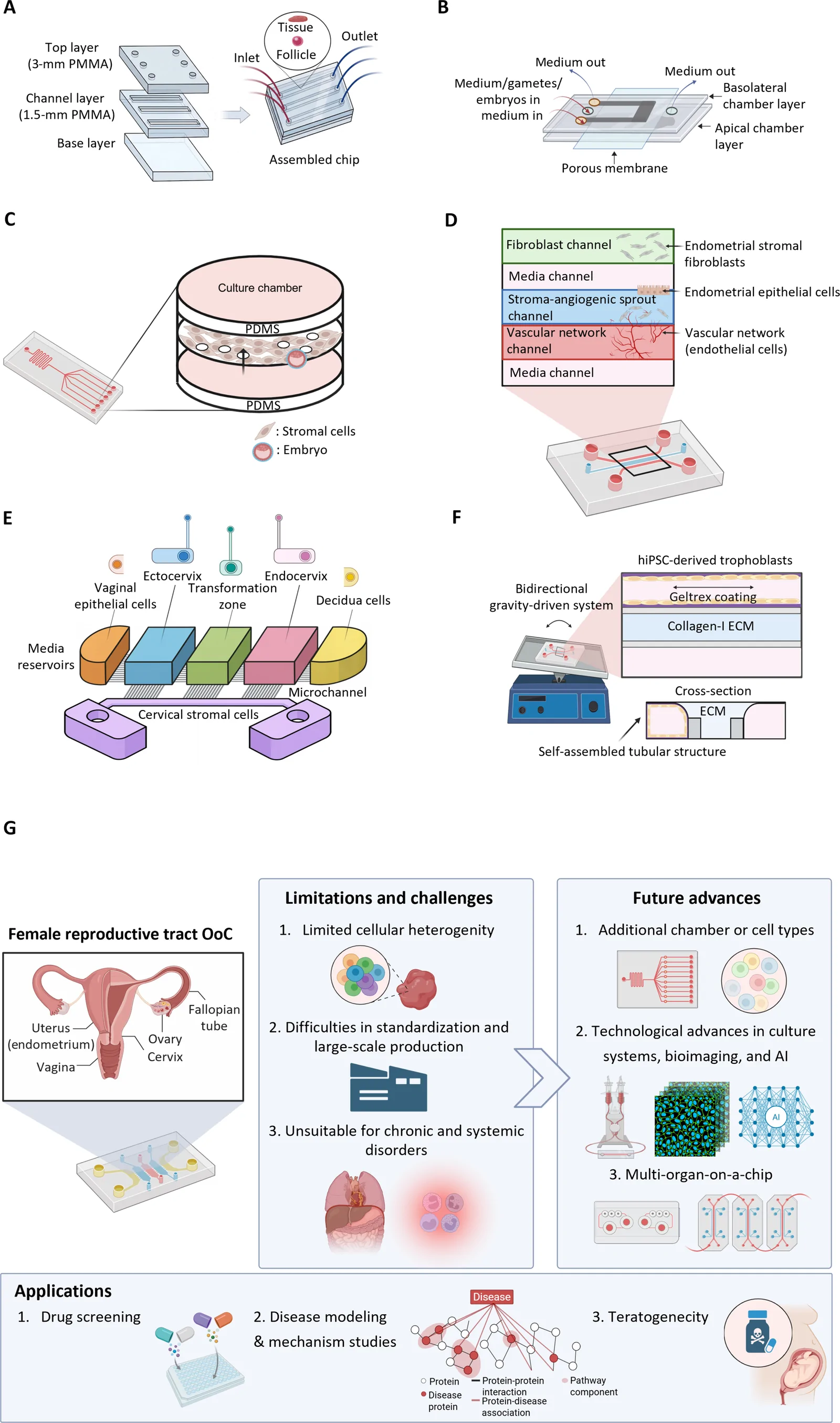
Schematic of female reproductive OoC systems and future advances in OoC technologies. (A) Configuration of the multi-layered ovary-on-a-chip. (B) Oviduct-on-a-chip incorporating a porous polycarbonate membrane between 2 poly-dimethylsiloxane (PDMS) layers. (C) Culture chamber of the uterus-on-a-chip for the coculture of embryo and stromal cells. (D) Endometrium-on-a-chip tri-culture model including endometrial stromal fibroblasts, endometrial epithelial cells, and endothelial cells. (E) Vagina-cervix-decidua-on-a-chip (VCD-OoC) device containing microchannels, cell chambers, and media reservoirs. (F) Illustration of the gravity-driven placenta-on-a-chip using the OrganoPlate platform, cultured with iPSC-derived trophoblasts. (G) Current OoC systems have limitations in cellular diversity, reproducibility, and systemic modeling. Advanced technologies including culture systems, bioimaging, and AI integration will enable enhanced applications to drug screening, disease modeling, and teratogenicity studies.

Microphysiological systems (OoC; Fig. 2A to F) that recapitulate female reproductive organs have attracted attention for their potential to improve embryo culture condition and subsequent outcomes in ART [61–64]. Historically, efforts to optimize in vitro embryo culture have concentrated on refining culture media formulations and supplements [65], along with fine-tuning physicochemical parameters such as oxygen tension, temperature, and pH [66–68]. Despite these advancements, both embryonic development efficiency and embryo competence remain suboptimal under conventional in vitro conditions.

### Ovary-on-a-chip for follicle culture

An in vitro platform capable of supporting the maturation of ovarian follicles into competent oocytes is essential for advancing fertility preservation. Compared with murine models, translation of in vitro follicle culture systems to larger mammals remains challenging due to the prolonged kinetics of folliculogenesis and the substantially larger follicular structures [69,70]. To overcome these limitations, Nagashima et al. [71] developed an ovary-on-a-chip platform that integrates microfluidic devices with either ovarian cortical tissue explants or isolated follicles from domestic cats and dogs (Fig. 2A). Compared with static culture, the microfluidic system elicited context-dependent follicular phenotypes, wherein follicles embedded within cortical tissue exhibited responses distinct from those of isolated follicles maintained under comparable conditions. These observations emphasize the importance of tissue context and dynamic perfusion in the design of translational platforms for in vitro follicle maturation.

### Fallopian tube-on-a-chip

**Fig. 3.**
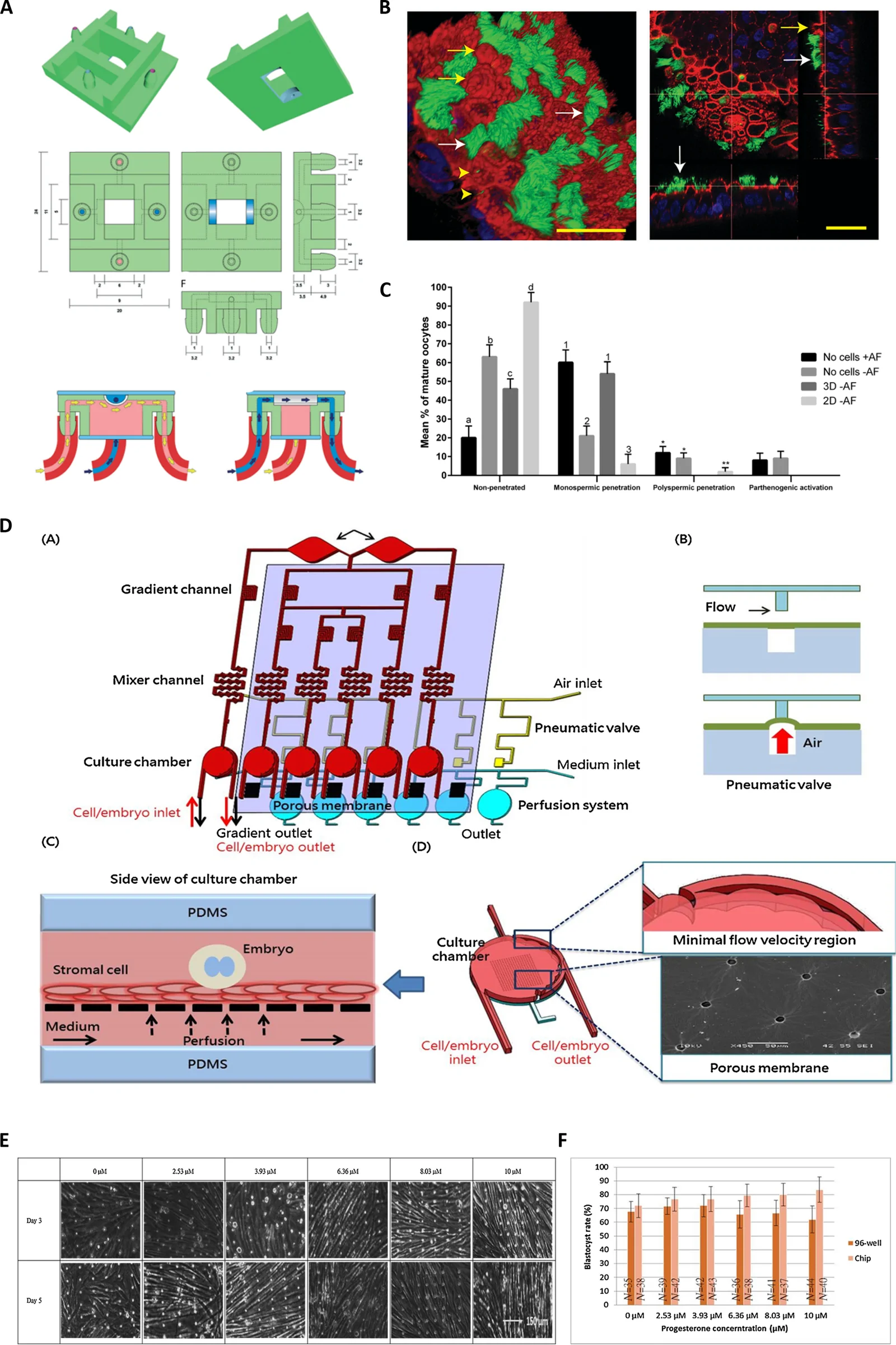
Microfluidic oviduct-on-a-chip characterization and biomimetic uterus-on-a-chip supporting embryo–endometrial interactions. (A) Schematic representation of the 3D-printed oviduct-on-a-chip device with separated apical and basolateral compartments. (B) Confocal images showing differentiated and polarized bovine oviduct epithelial cells with ciliated structures. (C) Functional assessment demonstrates comparable monospermic fertilization and reduced polyspermy and parthenogenetic activation compared to conventional in vitro fertilization systems. (D) Schematic of the uterus-on-a-chip device incorporating a concentration gradient generator, culture chamber, and perfusion system to mimic the uterine-like microenvironment. (E) Endometrial stromal cell responses to hormone stimulation, indicating the establishment of a physiologically relevant endometrial-like niche. (F) Enhanced blastocyst development under dynamic microfluidic conditions compared to conventional static culture systems. (A to C) Reproduced from Ferraz et al. [62], under the terms of the Creative Commons CC-BY 3.0 license. Copyright © 2017 The Authors. (D to F) Reproduced with permission from Chang et al. [79]. Copyright © 2015 Elsevier.

Kim et al. [61] introduced a mechanically active microfluidic in vitro cultivation platform that biomimetically recapitulates oviductal peristalsis observed in vivo. To assess the effect of mechanical stimulation on bovine embryo development within microfluidic channels, embryos were cultured either in straight channels or in channels with constrictions of varying widths. Using a micro-modulated tilting platform to generate controlled dynamic flow, comparative analyses further demonstrated that mechanical stimulation markedly enhanced early embryonic development. Because conventional culture systems often lead to a rapid loss of the features of BOECs, Ferraz et al. [62] established a physiologically relevant in vitro oviduct model that preserved epithelial polarization and differentiation, thereby enabling successful fertilization. The group also developed a bovine oviduct-on-a-chip featuring distinct apical and basolateral compartments that allowed hormonal modulation according to the estrous cycle, with integrated pillar arrays mimicking the physical entrapment of oocytes and embryos within the oviduct (Fig. 2B). A representative oviduct-on-a-chip platform incorporates a 3D microfluidic architecture with separated apical and basolateral compartments, enabling controlled fluid flow and cellular organization (Fig. 3A). Within this system, BOECs maintain their differentiated and polarized phenotype, including ciliated structures, closely resembling the in vivo oviductal epithelium (Fig. 3B). Functionally, the oviduct-on-a-chip supports physiologically relevant fertilization, significantly reducing polyspermy and parthenogenetic activation while maintaining efficient monospermic fertilization compared to conventional in vitro fertilization systems (Fig. 3C). These findings demonstrate the importance of recreating the oviductal microenvironment for improving fertilization outcomes in vitro. Zygotes generated in this system exhibited transcriptomic and methylation profiles more similar to in vivo embryos than those produced by standard in vitro embryo production (IVP) [63]. These features yield an in vivo-like environment that supports the generation of developmentally and physiologically relevant bovine zygotes. As another example of OoC platforms to mimic the female reproductive cycle, a mouse ovarian follicle-based microfluidic system was designed to mimic the human 28-d menstrual hormone profile, dynamically regulating interactions across single-, dual-, and multi-unit configurations [72]. The multi-unit platform, termed “EVATAR”, integrates 5 primary human cell types including cervical, fallopian tube, uterine, and hepatic cells, alongside mouse ovarian cells to model systemic endocrine crosstalk within the reproductive axis.

Since excessive reactive oxygen species (ROS) compromise genome integrity and epigenetic regulation, dynamically controlled systems are essential to minimize oxidative stress and improve developmental fidelity [73–75]. Wang et al. [64] demonstrated that dynamic microfluidic culture reduces intracellular ROS compared with static conditions. Using primary mouse oviduct epithelial cells, they further established a chip-based in vivo-like environment that minimized embryonic ROS burden and improved developmental outcomes during culture.

The OoC technology enables modeling of complex diseases and rare genetic disorders by recapitulating pathophysiology in organ-level dynamically controlled environments. Ferraz et al. [76] developed a microfluidic oviduct-on-a-chip using gene-edited canine oviduct epithelial cells to overcome limitations observed in in vivo rodent and 2D in vitro models for human high-grade serous carcinoma (HGSC)/serous tubal intraepithelial carcinoma (STIC). This system containing the apical and basolateral chambers mirrored the significant features of human STIC, including loss of cell polarization, reduced ciliation, and increased cell atypia and proliferation within multilayered epithelium. This model provides a platform for investigating STIC progression and discovering early HGSC biomarkers. In addition, building upon multi-unit EVATAR platform, Jackson-Bey et al. [77] established a fallopian tube-on-a-chip to investigate polycystic ovary syndrome (PCOS). Their study demonstrated that elevated testosterone levels disrupt ciliary activity and alter gene expression in human fallopian tube epithelial cells under both static and dynamic conditions, providing mechanistic insight into androgen-mediated reproductive dysfunction.

### Uterus-on-a-chip for embryo development

To address the higher incidence of birth defects associated with conventional in vitro fertilization, microfluidic chip-based systems are being developed to more accurately replicate the physiological in vivo reproductive environment. Li et al. [78] established a perfusable culture device allowing coculture of embryos and endometrial cells, thereby supporting sequential reproductive processes including ovulation, fertilization, implantation, and embryogenesis. Compared with static culture, this dynamic coculture system yielded higher rates of 4-cell, morula, and blastocyst formation, highlighting the importance of fluidic and cellular interactions in early embryonic development. Also, Chang et al. [79] developed a biomimetic microfluidic system that facilitates embryo coculture with endometrial stromal cells under continuous medium flow and mechanical stimulation (Fig. 2C). The device incorporates a gradient generator that supplies 6 distinct concentrations of progesterone to parallel culture chambers while maintaining a constant estrogen level. Semi-circular microstructures were incorporated to mimic ciliary-driven mechanical cues of the oviductal epithelium, promoting embryo development with temporal dynamics comparable to in vivo conditions (Fig. 3D). This platform mimics a physiologically relevant uterus-like microenvironment, as demonstrated by hormone-responsive proliferation of endometrial stromal cells (Fig. 3E). Importantly, dynamic microfluidic culture conditions enhance embryo development, resulting in increased blastocyst formation compared to conventional static culture systems (Fig. 3F). These findings highlight the importance of recreating the uterine microenvironment for improving in vitro embryo development.

**Fig. 4.**
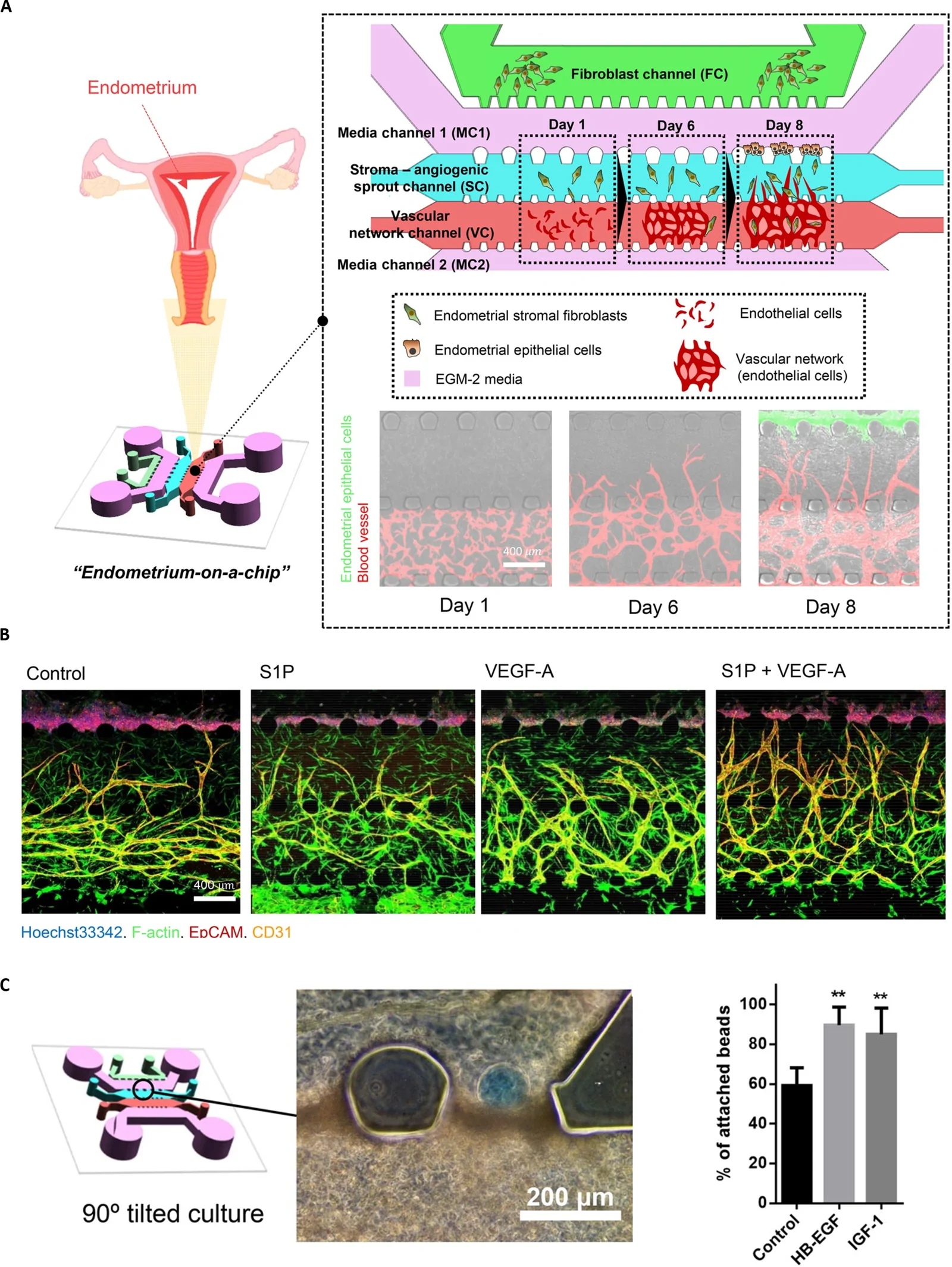
Vascularized endometrium-on-a-chip recapitulating implantation-related processes. (A) Schematic of a microengineered endometrium-on-a-chip integrating epithelial, stromal, and endothelial cell layers to mimic the endometrial microenvironment. (B) Formation of a microvascular network and angiogenic sprouting within the 3D extracellular matrix, demonstrating physiologically relevant vascularization. (C) Implantation model using ligand-coated microbeads, showing enhanced attachment to the endometrial epithelium and mimicking early embryo–endometrium interactions. Reproduced with permission from Ahn et al. [80]. Copyright © 2021 Oxford University Press.

Recently, beyond simply reproducing the multilayered architecture and vascular complexity of the human endometrium, a microengineered vascularized endometrium-on-a-chip (MVEOC) was established (Fig. 2D), which integrates epithelial, stromal, and vascular layers (Fig. 4A). In coculture with endometrial stromal fibroblasts, endothelial cells within the vascular compartment formed vasculatures responsive to vascular endothelial growth factor-A (VEGF-A) (Fig. 4B). Moreover, by introducing microbeads functionalized with heparin-binding EGF-like growth factor (HB-EGF) and insulin-like growth factor I (IGF-I) to mimic embryo adhesion (Fig. 4C), the MVEOC served as a platform to investigate implantation dynamics and early human pregnancy mechanisms [80].

### Cervix-on-a-chip

**Fig. 5.**
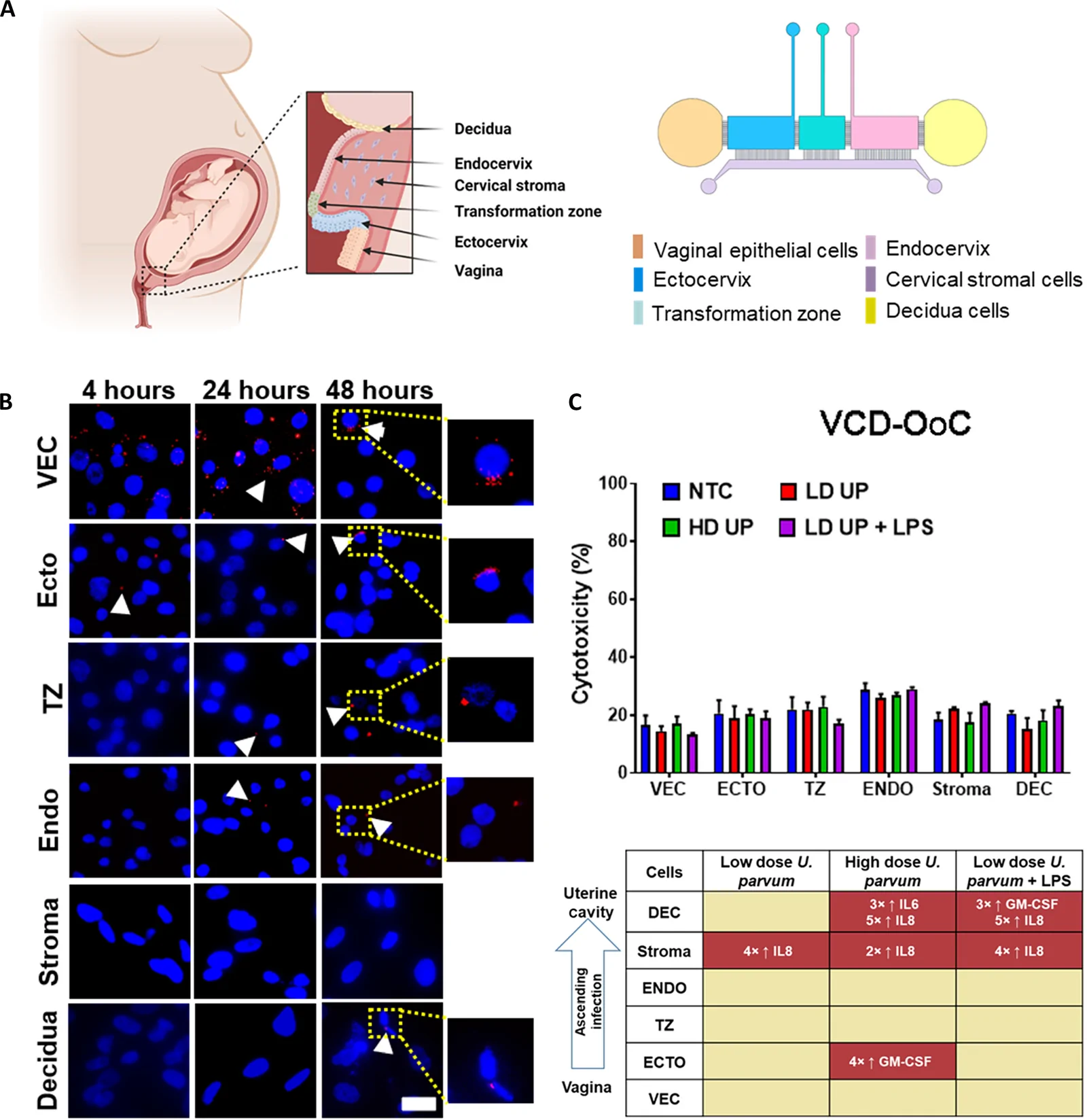
Vagina–cervix–decidua–OoC (VCD-OoC) model recapitulating ascending infection and barrier properties. (A) Schematic of a multi-compartment VCD-OoC integrating epithelial and stromal layers to mimic the lower FRT. (B) Time-dependent propagation of *U. parvum* from the vaginal epithelial compartment to cervical and decidual layers, demonstrating ascending infection. (C) Inflammatory responses induced by infection, showing cytokine production across different cell layers under bacterial and polymicrobial conditions. Reproduced with permission from Tantengco et al. [82]. Copyright © 2022 John Wiley and Sons.

As the cervix functions as a key barrier against ascending infections, its dysfunction can cause preterm birth, infertility, and bacterial vaginosis, highlighting the need for physiologically relevant in vitro models to effectively study these pathophysiological defects. Tantengco et al. [81] first reported the cervical epithelial layer-organ-on-chip (CE-OoC) comprising distinct ectocervical and endocervical epithelial compartments interconnected through microfluidic channels. The platform successfully recapitulated inflammatory responses to external stimuli such as lipopolysaccharide (LPS) and tumor necrosis factor-α (TNF-α), evidenced by elevated cytokine expression and epithelial–mesenchymal transition (EMT). The limited physiological relevance of the CE-OoC due to the absence of stromal and immune components led to the development of the vagina–cervix–decidua–on-a-chip (VCD-OoC), which incorporates 6 representative cell types from vaginal, cervical, and decidual tissues (Fig. 2E) [82]. Incorporation of stromal cells enabled more physiologically relevant cell–cell interactions across epithelial and stromal compartments. Importantly, this platform allows the modeling of ascending infection across the cervicovaginal interface, where pathogens introduced into the vaginal compartment propagate through cervical epithelial layers and reach the decidual compartment in a time-dependent manner (Fig. 5A and B). Furthermore, the system captures cell-type-specific inflammatory responses, demonstrating differential cytokine production primarily in stromal and decidual compartments, with minimal inflammatory responses in epithelial layers, under infectious conditions (Fig. 5C). Conditioned media from the maternal human fetal membrane-decidual cell chamber of the VCD-OoC were subsequently applied to a feto-maternal interface–on-a-chip (FMi-OoC) to further investigate the impact of *Ureaplasma parvum* infection on fetal tissues. Izadifar et al. [83] introduced a microfluidic cervical model that reproduces key physiological functions, such as mucus production, microbial interactions, and hormone responsiveness. Although the absence of immune and endothelial constituents still constrains completeness, the platform represents a significant step toward physiologically relevant systems for investigating host–microbiome interactions and testing therapeutic strategies. Given that male infertility is frequently associated with abnormal sperm morphology, low cellular concentration, reduced motility, and DNA damage, reconstitution of the cervical mucus layer, which naturally supports sperm survival and selective passage, may aid in selecting functionally competent sperm to improve ART outcomes [84,85]. In the line, Lee et al. [86] created a polyvinylpyrrolidone (PVP)-based microfluidic sperm-sorting chip that replicates a viscoelastic cervical environment, yielding sperm with higher DNA integrity and offering a gentler alternative to conventional swim-up and density-gradient centrifugation techniques.

### Placenta-on-a-chip

**Fig. 6.**
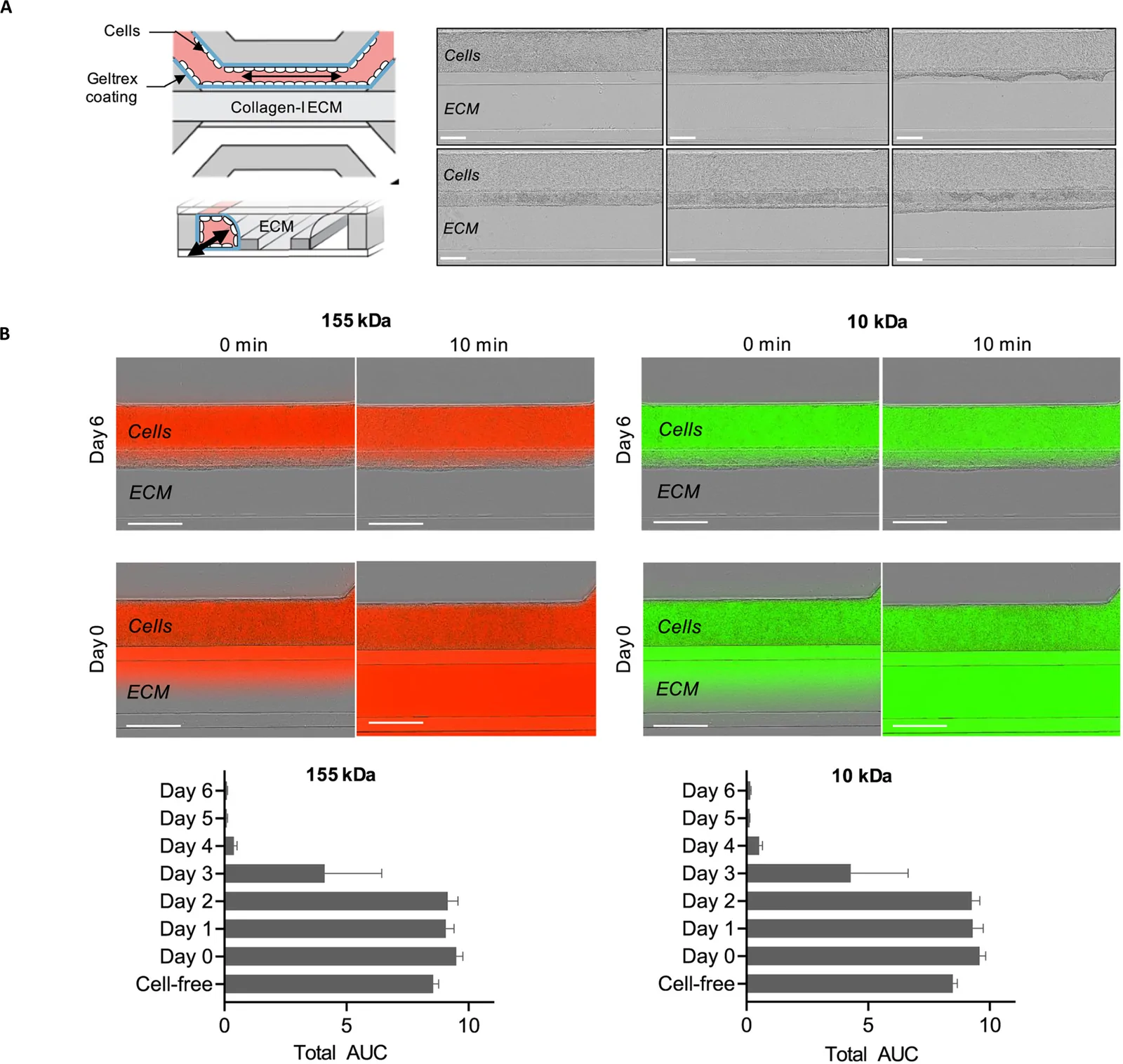
Placenta-on-a-chip model recapitulating the maternal–fetal barrier. (A) Schematic of a microfluidic placenta-on-a-chip platform showing ECM-supported microchannels and trophoblast barrier formation. (B) By day 6 of differentiation, placental barrier exhibits integrity, preventing the leakage of molecules. Reproduced from Lermant et al. [89], under the terms of the Creative Common CC BY 4.0 license. Copyright © 2023 The Authors. Published by Elsevier.

To model placental functions of nutrient and gas exchange and fetal protection, Cao et al. [87] cocultured human trophoblast stem cells (hTSCs) and endothelial cells to construct a placenta-on-a-chip platform that recapitulates the placental barrier. hTSCs within the construct differentiated toward trophoblast lineages including CTBs and STBs, forming a bilayered epithelium densely packed with microvilli. Under fluidic conditions, key placental functions such as hCG secretion, lipid metabolism, and glucose transport were enhanced, accompanied by Rap1 pathway activation associated with trophoblast syncytialization [87]. Moreover, exposure to the environmental toxin such as mono-2-ethylhexyl phthalate (MEHP) induced distinct toxicological responses in the platform, demonstrating applicability for assessing chemical toxicity during early pregnancy. To present trophoblast lineage development more precisely, subsequent placenta-on-a-chip models incorporated human induced pluripotent stem cells (hiPSCs), enabling the generation of the major trophoblast subtypes, CTBs, STBs, and EVTs, while fluidic stimulation activated calcium and mitogen-activated protein kinase (MAPK) signaling pathways [88]. Especially, up-regulation of ERVW-1, CDH5, and ZO-1 under dynamic perfusion enhanced tight-junction formation and shear stress-mediated signaling, resulting in larger and more physiologically representative 3D placental structures compared to static cultures. For the scalability and multiplex throughput, a multi-chip OrganoPlate system was introduced, as an alternative to single-unit device, in which hiPSCs self-organized into hollow tubular structures that differentiated into CTBs and STBs to form a leak-tight barrier (Fig. 2F). The system also up-regulated key transporter genes, supporting functional maternal–fetal molecular exchange [89]. To further characterize the functional properties of this system, barrier integrity was evaluated using fluorescent tracers. A comparison between day 0 and day 6 demonstrates the formation of leak-tight barrier, which restricts the transport of both small and large molecules (Fig. 6A and B). Yet, current placenta-on-a-chip models do not fully recapitulate placental complexity, highlighting the need for next-generation systems capable of higher-order tissue organization and maternal–fetal interactions.

## Challenges and Future Advances in OoC

OoC systems can recapitulate key physiological features of human organs and provide human-relevant alternatives to conventional animal models. However, several limitations remain. First, current FRT OoC models do not fully reproduce the cellular heterogeneity of native tissues. Because FRT physiology depends on dynamic molecular signaling and interactions among diverse cell populations, models composed of only a few cell types cannot adequately capture its complexity. Future systems should therefore incorporate additional cell types, including immune and endothelial cells, through multicellular coculture or additional compartments [83,89]. Second, broader translational adoption remains challenging due to difficulties in standardization, scalability, and batch-to-batch reproducibility. A scalable, pump-free flow device with automated fluid control has been developed for barrier-modeling OoC platforms and applied to skin, blood–brain barrier, and lung models [90]. Adaptation of this approach to the FRT could support high-throughput analysis while preserving barrier-specific properties. However, pump-free systems may be less suitable for models requiring precisely controlled unidirectional flow or complex multicellular organization. Further advances in culture design, bioimaging, and machine learning-based analysis may improve reproducibility and facilitate standardized data interpretation [91]. Finally, current OoC systems have limitations in modeling chronic and systemic inflammatory disorders. Most FRT OoC platforms represent a single organ and lack immune components and inter-organ interactions involving the liver, kidney, heart, and other tissues. Multi-OoC systems have been developed to address this limitation, but they still face challenges related to long-term maintenance and the limited number of organs that can be integrated [92]. Future reproductive OoC platforms should therefore combine optimized multicompartmental designs with immune and multi-organ components to model systemic responses more effectively. Such advances could expand their applications in disease-mechanism studies, drug screening, and the evaluation of anti-inflammatory, antimicrobial, and teratogenic effects (Fig. 2G).

### 3D-based NAMs

To overcome the limitations of animal models and conventional 2D culture systems, 3D-based NAMs, including organoids and OoC systems, have emerged as human-relevant platforms for reproductive biology research. These models better reproduce tissue architecture, cell–cell and cell–matrix interactions, cellular heterogeneity, and microenvironmental cues than 2D monolayers [93]. 3D NAMs offer controlled manipulation of cellular composition, matrix properties, biochemical stimuli, oxygen gradients, hormone exposure, and fluid flow, thereby supporting mechanistic investigation, target validation, disease modeling, and therapeutic testing [94]. Particularly in drug development, these platforms provide human-relevant cellular and tissue microenvironments that facilitate candidate screening, lead optimization, toxicity assessment, and patient-specific therapeutic evaluation, thereby improving the early prediction of efficacy and safety [93]. The translational value of 3D NAMs is further strengthened by advances in validation, reproducibility, and assay standardization. Validation evaluates whether a model is fit for a defined context of use, such as disease modeling, toxicity testing, barrier assessment, or drug screening. Defining the intended context of use, together with standardized cell sources, culture conditions, exposure protocols, functional endpoints, and controls, is essential for establishing these models as reliable preclinical tools rather than descriptive in vitro systems [95].

Organoids and OoC systems constitute complementary 3D NAM platforms for modeling human physiology. Organoids, self-organizing 3D structures derived from stem cells or patient-derived tissues, preserve tissue-specific organization, lineage characteristics, and disease-associated phenotypes, supporting patient-specific disease modeling, drug-response prediction, and personalized therapeutic evaluation. Their compatibility with multiwell plate-based formats further enables scalable and systematic screening across diverse biological samples and treatment conditions [96]. In contrast, OoC systems offer enhanced physiological relevance by incorporating microfluidic flow, mechanical stimulation, tissue–tissue interfaces, and biochemical gradients. These engineering features allow OoC platforms to model functional tissue responses under controlled conditions that more closely resemble in vivo microenvironments. Their compartmentalized architectures enable functional assessment of barrier integrity, epithelial–stromal interactions, drug transport, and organ–organ communication [97]. From a translational perspective, organoids and OoC systems provide complementary capabilities in preclinical research. Organoids facilitate patient-specific modeling and scalable screening, whereas OoC platforms enable dynamic physiological regulation and functional assessment under controlled microenvironmental conditions [96]. Their combined application may therefore improve the physiological relevance and predictive performance of human in vitro models for drug development and therapeutic evaluation.

## Conclusion and Future Perspectives

Since conventional 2D culture methods face difficulties in maintaining the characteristics of epithelial cells for long-term in vitro, various 3D culture methods have been developed including ALI, spheroid, organoid, and reproductive OoC systems, which more closely mimic in vivo microenvironment. In particular, fluidic oviduct-on-a-chip systems have enabled controlled investigation of ciliary function, gamete–embryo interactions, hormonal responses, and early developmental events within a near-physiological microenvironment. Organoids derived from ovary, fallopian tube, uterus, cervix, and placenta similarly provide stable, lineage-relevant platforms for studying development, disease mechanisms, and individual physiology. Collectively, these advances represent key achievements in the development of human-relevant models for investigating female reproductive physiology and disease.

Organoids and OoC systems provide distinct but complementary capabilities. Organoids preserve tissue-specific organization, lineage identity, and patient-derived characteristics, supporting disease modeling, drug-response prediction, and scalable screening [93]. In contrast, OoC systems provide precise control over fluid flow, mechanical stimulation, biochemical gradients, and tissue–tissue interfaces, enabling functional analyses under dynamic physiological conditions [8,93]. Their integration may combine the biological complexity of organoids with the controlled microenvironment and functional readouts of OoC platforms, thereby improving the physiological relevance and predictive capacity of reproductive models [60,93].

Despite these promising developments, notable technical and biological challenges remain. One of the key obstacles is maintaining long-term differentiated functionality of epithelial cells as they rapidly lose the characteristics under in vitro culture conditions. Additionally, reproducing cyclic hormonal rhythms that drive ovulation, fertilization, embryo transport, and endometrial receptivity is technically challenging. Most organoid systems also lack stromal, immune, endothelial, and neural components, limiting their ability to reconstruct tissue–tissue interactions. Many reproductive OoC models incorporate only a limited number of cell types and therefore do not fully reproduce native tissue heterogeneity. Variability in cell sources, matrices, and assay conditions further limits reproducibility and translational use.

While polydimethylsiloxane (PDMS) is prevalently employed for the fabrication of OoC systems, particularly for drug testing, it can be another significant challenge due to its property to absorb small-molecule compounds, which affects the accuracy of drug or hormone assays [98,99]. Thus, the identification of alternative materials and the development of advanced microfabrication techniques are required to ensure more reliable outcomes. Lastly, improving the scalability and reproducibility of these systems will be crucial for their broader application in clinical research and drug testing. The use of chemically defined or synthetic matrices, together with standardized cell sources, culture conditions, endpoints, and analytical criteria, will be essential for improving platform reliability and translational applicability.

As a next step, integrative reproductive axis-on-a-chip systems have to be developed to create a comprehensive, multi-organ reproductive tract chip. Such platforms will provide unprecedented opportunities to interrogate infertility, ectopic pregnancy, early pregnancy loss, implantation failure, and endocrine disorders [8]. For instance, earlier mentioned EVATAR is a multi-OoC that replicates the female reproductive system and liver on a single chip, enabling the simulation of liver metabolism for pharmacological and toxicological testing related to the female reproductive system. Current multi-organ platforms remain limited by challenges in long-term maintenance, inter-organ compatibility, and the number of tissues that can be integrated. Future systems should incorporate additional reproductive, immune, hepatic, and vascular components to better model systemic responses. Organoid and OoC technologies are increasingly enabling precise recapitulation of native tissue architecture and disease pathophysiology across a wide range of organ systems, reflecting a broader and ongoing transformation in how biological complexity is modeled in vitro. Such progresses are not confined to reproductive biology alone; brain organoids recapitulating human neural architecture for disease modeling and drug screening [100], as well as bone marrow (BM) organoids and BM-on-a-chip systems replacing murine xenograft models for hematopoietic research [101], collectively reflect a paradigm shift toward human-centric, biomimetic platforms that is actively reshaping biomedical research across organ systems.

The integration of reproductive organoids and OoC systems with single-cell and spatial omics, computational modeling, and artificial intelligence (AI) represents another important future direction. Single-cell and spatial analyses can resolve cellular heterogeneity and identify lineage-specific responses that are not detectable using conventional bulk measurements. Computational models can support the quantitative analysis of hormone dynamics, fluidic transport, drug distribution, and inter-organ interactions [72], whereas AI-driven approaches may facilitate image-based phenotyping, quality control, dose–response analysis, and standardized data interpretation. These integrated approaches could improve the prediction of differentiation trajectories, disease progression, therapeutic efficacy, and toxicity. Ultimately, scalable reproductive organoid and OoC platforms incorporating defined matrices, multicellular complexity, dynamic hormonal regulation, omics, and computational analysis could reduce reliance on 2D cultures and animal models while improving disease modeling, therapeutic evaluation, and reproductive health research.

## Data Availability

Not applicable. This study did not generate any new data.
