# Supplementary material for: Female Reproductive Tract Modeling through Advanced 3D Biomimetic Platforms
Source: Biomater Res. 2026 Sep 3;30:0413. doi: 10.34133/bmr.0413 (PMC13538936; doi:10.34133/bmr.0413)
Supplement: Supplementary file 1 [file bmr.0413.f1.docx]

**[Supplementary Materials]**

**Female Reproductive Tract Modeling through Advanced 3D Biomimetic Platforms**

**Authors**

Chaeyoun Lee^1,2, *^, Yoonseo Choi^1,2, *^, Hyejin Lee^1,2^, Seunghee Kim^1,2^, Jeong Su Park^3^, Young Min Shin^1,2^, Dae Hyeok Yang^4^, Gun-Jae Jeong^5^, Dong Nyoung Heo^6^, Jeong-Kee Yoon^7^, Junhyeong Yim^1,2^, Jongmin Kim^1,2^, Yonghwan Kim^1,2^, Kyung Hyun Yoo^1,2^, Jung Bok Lee^1,2, †^, Byung-Chul Lee^1,2,†^

**Affiliation**

^1^Department of Biological Sciences, Sookmyung Women’s University, Seoul 04312, Republic of Korea.

^2^Research Institute of Women’s Health, Sookmyung Women’s University, Seoul 04312, Republic of Korea.

^3^PLCOskin Co., Ltd., Seoul 03721, Republic of Korea.

^4^Department of Biologics and Engineering, The Catholic University of Korea, Bucheon-si, 14662, Gyeonggi-do, Republic of Korea

^5^School of Nanomedical Engineering, Korea National University of Transportation, Chungju 27469, Republic of Korea.

^6^Department of Dental Materials, School of Dentistry, Kyung Hee University, Seoul, 02447, Republic of Korea.

^7^Department of Systems Biotechnology, Chung-Ang University, Anseong-Si, Gyeonggi-Do, 17546, Republic of Korea.

^*^These authors contributed equally to this work

^†^Correspondence;

**Jung Bok Lee, PhD**

Department of Biological Sciences

Research Institute of Women’s Health,

Sookmyung Women’s University, Seoul, Korea

Phone: +82-2-710-9486

E-mail: [jungboklee@sookmyung.ac.kr](mailto:jungboklee@sookmyung.ac.kr)

**Byung-Chul Lee, DVM, PhD**

Department of Biological Sciences

Research Institute of Women’s Health,

Sookmyung Women’s University, Seoul, Korea

Phone: +82-2-2077-7252

E-mail: [bclee@sookmyung.ac.kr](mailto:bclee@sookmyung.ac.kr)

**Supplementary Figures**

**Figure S1. Generation of reproductive organoids** Organoids are generated from tissue-resident cells or pluripotent stem cells and self-organize into three-dimensional structures that preserve epithelial identity and tissue architecture.
